# Flow Halted to Save Life: Functional Annulment of the Distal Right Coronary Artery Using a Covered Stent for Refractory Perforation During Chronic Total Occlusion Intervention

**DOI:** 10.7759/cureus.97739

**Published:** 2025-11-25

**Authors:** Ankit Gupta, Ojas Gowda S G, Zainab Mehdi, Bhushan Shah, Ashutosh P Tripathi, Sreenivas Reddy

**Affiliations:** 1 Cardiology, All India Institute of Medical Sciences, Raebareli, Raebareli, IND; 2 Internal Medicine, All India Institute of Medical Sciences, Raebareli, Raebareli, IND; 3 General Medicine, Emergency Medicine, Government Medical College and Hospital, Chandigarh, Chandigarh, IND; 4 Cardiology, All India Institute of Medical Sciences, Bhopal, Bhopal, IND; 5 Cardiology, All India Institute of Medical Sciences, Gorakhpur, Gorakhpur, IND; 6 Cardiology, Government Medical College and Hospital, Chandigarh, Chandigarh, IND

**Keywords:** acute right coronary artery occlusion, chronic total occlusion, coronary artery perforation (cap), inferior wall myocardial infarction, targeted annulment

## Abstract

Percutaneous coronary intervention (PCI) is a minimally invasive treatment that reestablishes the flow in occluded coronary arteries with the use of wires, balloons, and stents. Chronic total occlusion (CTO) PCIs are one of the most challenging procedures in interventional cardiology because the morphology of the lesions is complex, the duration is prolonged, and there is a higher risk of complications like vessel perforation. The management of distal perforations can sometimes be very problematic when conventional approaches have failed.

A 47-year-old male was admitted with acute chest pain and diagnosed with an inferior wall myocardial infarction. Coronary angiography revealed a CTO of the right coronary artery (RCA). Standard CTO techniques, including guidewire escalation, microcatheter support, sequential balloon angioplasty, and deployment of a drug-eluting stent (DES), were initiated to perform PCI. During the procedure, distal RCA perforation occurred. Initial management with prolonged balloon tamponade was unsuccessful, and a covered stent could not be advanced due to the tortuosity of the vessel. To address this, an alternative approach was employed whereby access was obtained via the marginal branch by dilating the stent struts of the previously deployed DES, allowing the covered stent to be advanced and deployed into the acute marginal branch, effectively isolating the perforated segment.

This case illustrates a novel bailout approach for managing distal RCA perforation during complex CTO-PCI. When the delivery of a covered stent is not possible via a standard approach, accessing the main vessel through a side branch and stent strut dilatation may offer a viable route to successful sealing of perforation.

To the best of our knowledge, this is the first case in the literature of distal RCA perforation that was successfully managed by functional annulment using a covered stent delivered through a side branch after stent strut dilatation. This technique illustrates a creative and effective solution for a rare but potentially catastrophic complication.

The patient remained hemodynamically stable post-procedure. Serial echocardiography demonstrated no pericardial effusion, and cardiac biomarkers appropriately trended down. The patient was asymptomatic at the three-month follow-up with preserved left ventricular function.

## Introduction

Percutaneous coronary intervention (PCI) in chronic total occlusion (CTO) remains a high-stakes procedure in interventional cardiology, often associated with increased technical difficulty and risk [1]. Coronary artery perforation (CAP) is rare but life-threatening. It is further classified into proximal artery, distal artery, and epicardial artery perforation. CAP occurs in approximately 0.2-0.5% of all PCI cases, with higher rates in complex procedures such as CTO interventions. Major risk factors include female sex, hypertension, chronic kidney disease, prior bypass grafting, complex lesion anatomy, and use of advanced PCI techniques or devices [2-4]. Mortality rates have declined over time, now averaging around 7.5%, with cardiac tamponade occurring in about 20% of cases [2,4-8].

Distal artery perforation commonly occurs in old, calcified, and complex lesion anatomy due to guidewire manipulation [3]. They are mainly managed by percutaneous interventions, and in refractory cases, it is surgical. Around 12-21% of cases end up with surgical intervention due to failed percutaneous management or ongoing hemodynamic instability [2,4-8].

We present a case of distal right coronary artery (RCA) perforation in which prolonged balloon tamponade and anticoagulation reversal were unsuccessful, and a covered stent could not be delivered to the perforation site due to complex lesion anatomy. A novel approach was therefore employed, in which a covered stent was deployed into the acute marginal branch to functionally occlude the distal RCA, successfully controlling bleeding and stabilizing the patient.

## Case presentation

Echocardiography

**Figure 1:**
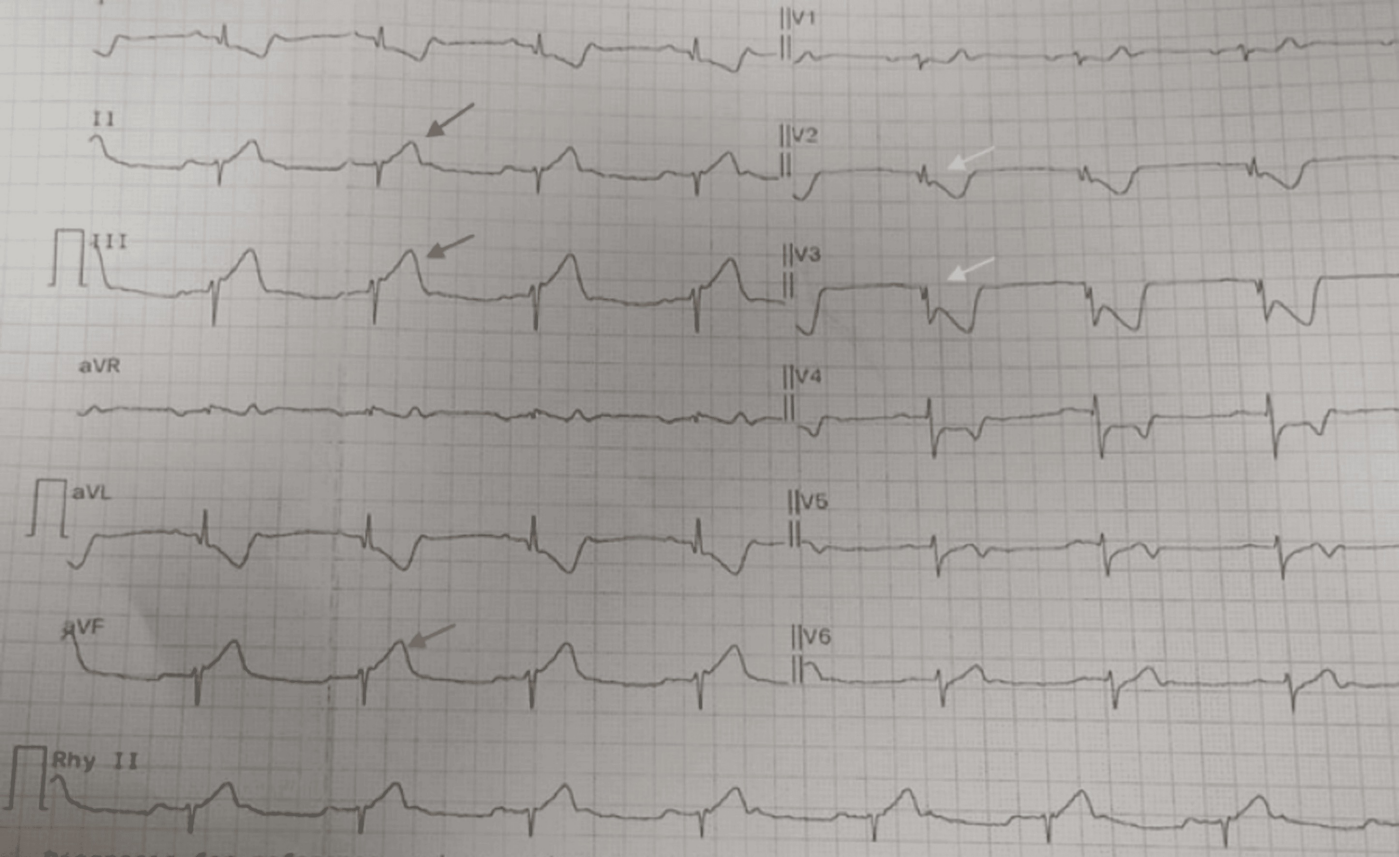
Baseline electrocardiogram 12-lead ECG at presentation Showing ST-segment elevation in inferior leads (III > II, aVF) (gray arrows) with reciprocal ST depression in V1-V4 (white arrows) consistent with acute inferior wall ST-elevation myocardial infarction (STEMI).

A 47-year-old male presented to the emergency department with retrosternal chest discomfort radiating to the left shoulder, with sweating and palpitations for the past 24 hours. He presented to the emergency department with vitals of 52 beats per minute heart rate, blood pressure of 112/80 mmHg, with a respiratory rate of 18/min, and maintaining saturation of 99% on room air. A bedside electrocardiogram was done, suggestive of an inferior wall myocardial infarction (Figure 1).

Upon initial evaluation

Laboratory profile: Cardiac biomarkers showed elevated high-sensitivity troponin I levels, confirming myocardial infarction. Complete blood count, renal function, electrolytes, and coagulation profile were within normal limits. Echocardiography showed a regional wall motion abnormality in the inferior wall.

Procedural details

**Figure 2:**
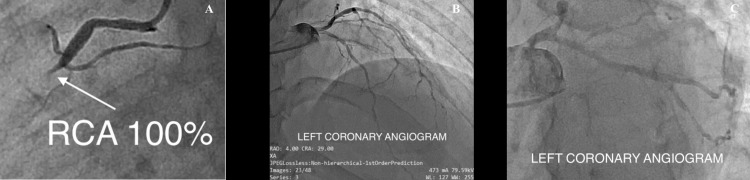
Coronary angiography Pre-percutaneous coronary intervention (PCI) diagnostic coronary angiogram showing total occlusion of the mid coronary artery (right coronary artery) with normal left circumflex (LCx) and left anterior descending (LAD). A: right coronary artery angiogram, showing chronic total occlusion (CTO) (white arrow); B, C: normal flow in the left coronary artery with Rentrop 1 collateral circulation.

Coronary angiography revealed a CTO of the mid-RCA (Figure 2) with thrombolysis in myocardial infarction (TIMI) grade 0 flow, and Rentrop 1 collateral circulation [9,10] can be seen in the left coronary angiogram (Figure 2).

Vascular access and guide engagement

**Figure 3:**
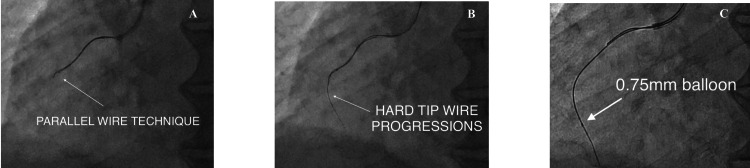
A Fielder XT wire with microcatheter support successfully reached the posterior descending artery (PDA) A: wiring the right coronary artery with Asahi Fielder XT wire (ASAHI Intecc Co., Ltd., Aichi, Japan) (white arrow); B: hard tip wiring was done to cross the chronic total occlusion (CTO) (white arrow); C: after crossing the lesion, a 0.75 mm balloon was inflated (white arrow).

PCI was initiated using a 6F JR 3.5 guiding catheter. Multiple guidewire strategies were employed, and an Asahi Sion Blue wire (ASAHI Intecc Co., Ltd., Aichi, Japan) failed to cross the lesion. An Asahi Fielder XT wire (ASAHI Intecc Co., Ltd., Aichi, Japan) with microcatheter support successfully reached the posterior descending artery (PDA), bypassing the mid CTO (Figure 3).

Wire exchange

**Figure 4:**
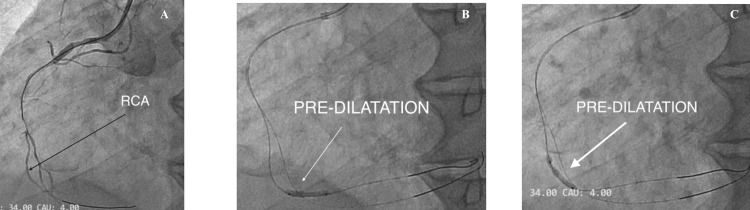
The wire was exchanged for a Sion Blue, and a second wire was positioned in the posterolateral ventricular (PLV) branch A: Fielder was exchanged with an Asahi Sion Blue wire (ASAHI Intecc Co., Ltd., Aichi, Japan) (black arrow); B, C: lesion was prepared by pre-dilatation serially (white arrow). RCA: right coronary artery

The wire was exchanged for a Sion Blue, and a second wire was positioned in the posterolateral ventricular (PLV) branch (Figure 4).

Lesion preparation

**Figure 5:**
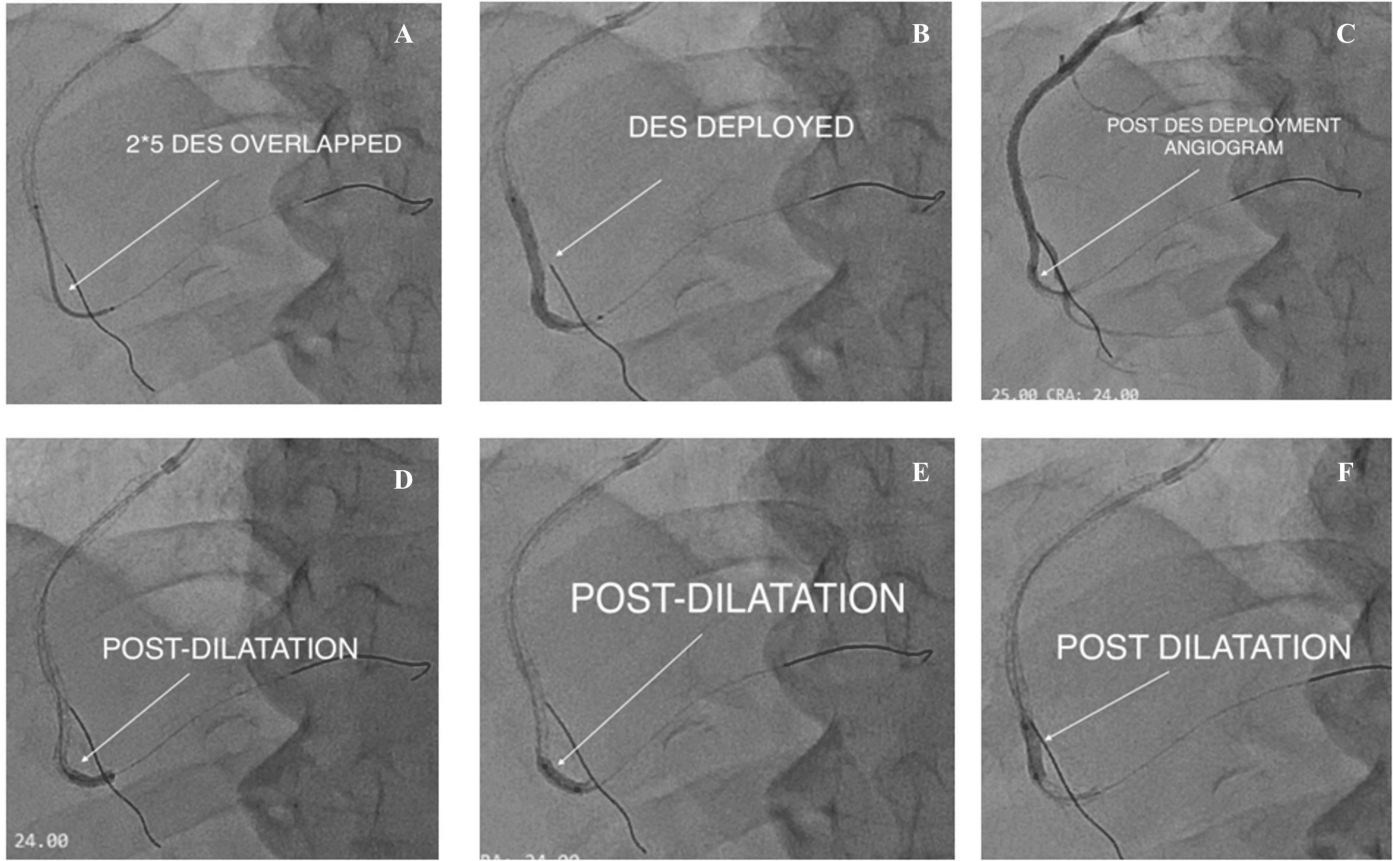
Pre- and post-dilatation were done, and a drug-eluting stent was deployed at the lesion site A: after preparing the lesion, a serial pre-dilatation drug-eluting stent was sent (white arrow); B: white arrow shows stent deployment; C: post-stent deployment angiogram (white arrow); D, E, F: all show post-deployment dilatation serially (white arrow).

Lesion preparation and stenting were initiated. Pre-dilatation was done with an Abbott TREK (Abbott, IL, USA) 1.2 × 8 mm balloon (8-10 atm) stenting, and the stent was deployed proximal to mid-RCA (12 atm). Post-dilatation was done with non-compliant (NC) TREK 2.75 × 12 mm (distal 12-16 atm; proximal 16-20 atm), and the second drug-eluting stent (DES) (2.5 × 40 mm Tetriflex (Sahajanand Medical Technologies Ltd (SMT), Gujarat, India)) was placed distally, and the final position was checked by angiography (Figure 5).

Complication and management

**Figure 6:**
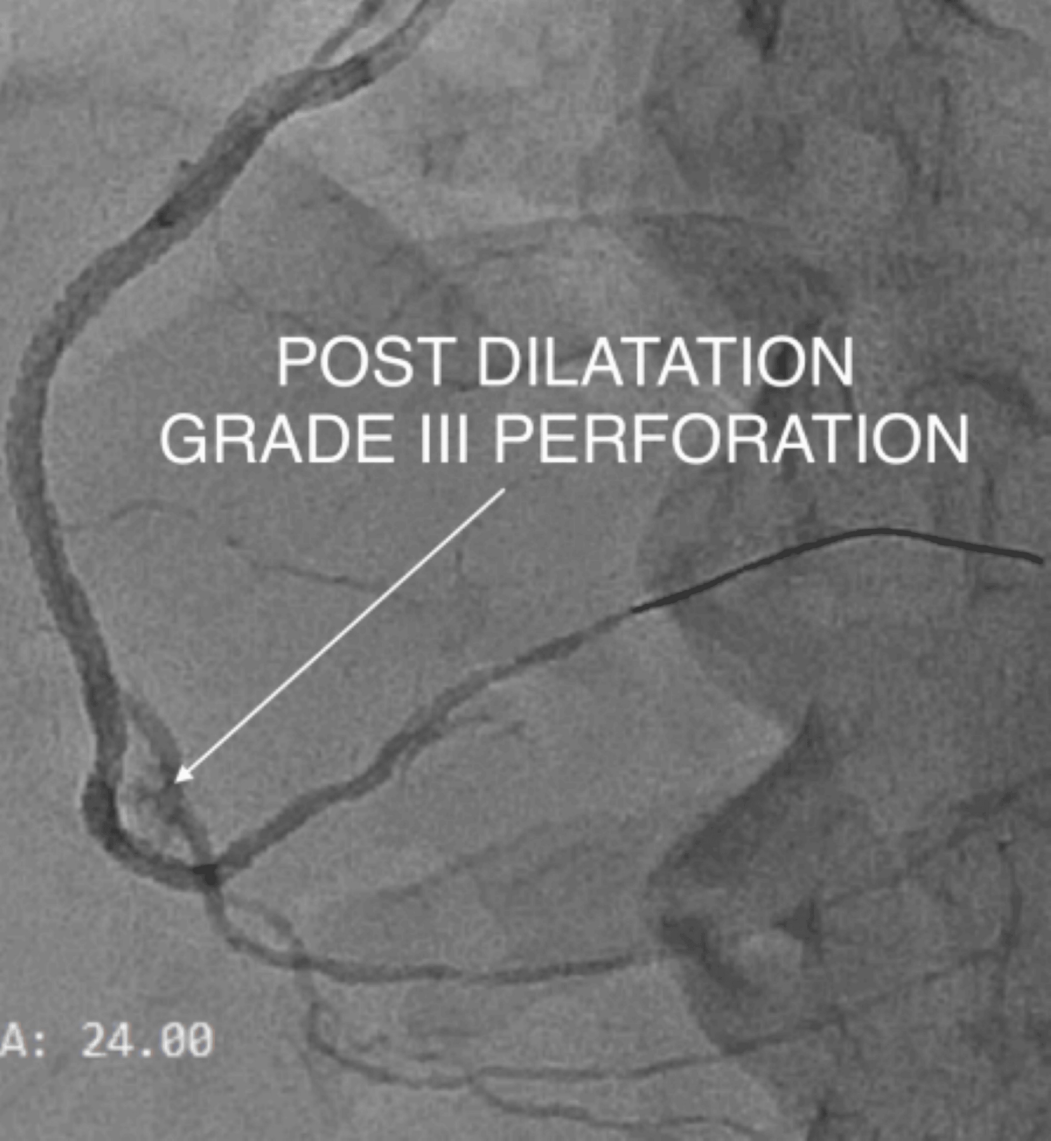
Post-procedure details On checking the contrast flow, it revealed a distal right coronary artery (RCA) perforation with an Ellis grade III type of perforation (white arrow).

Angiography revealed contrast extravasation consistent with a distal coronary perforation at the second bend of the RCA, corresponding to an Ellis type III injury (Figure 6) [11].

Immediate management measures

**Figure 7:**
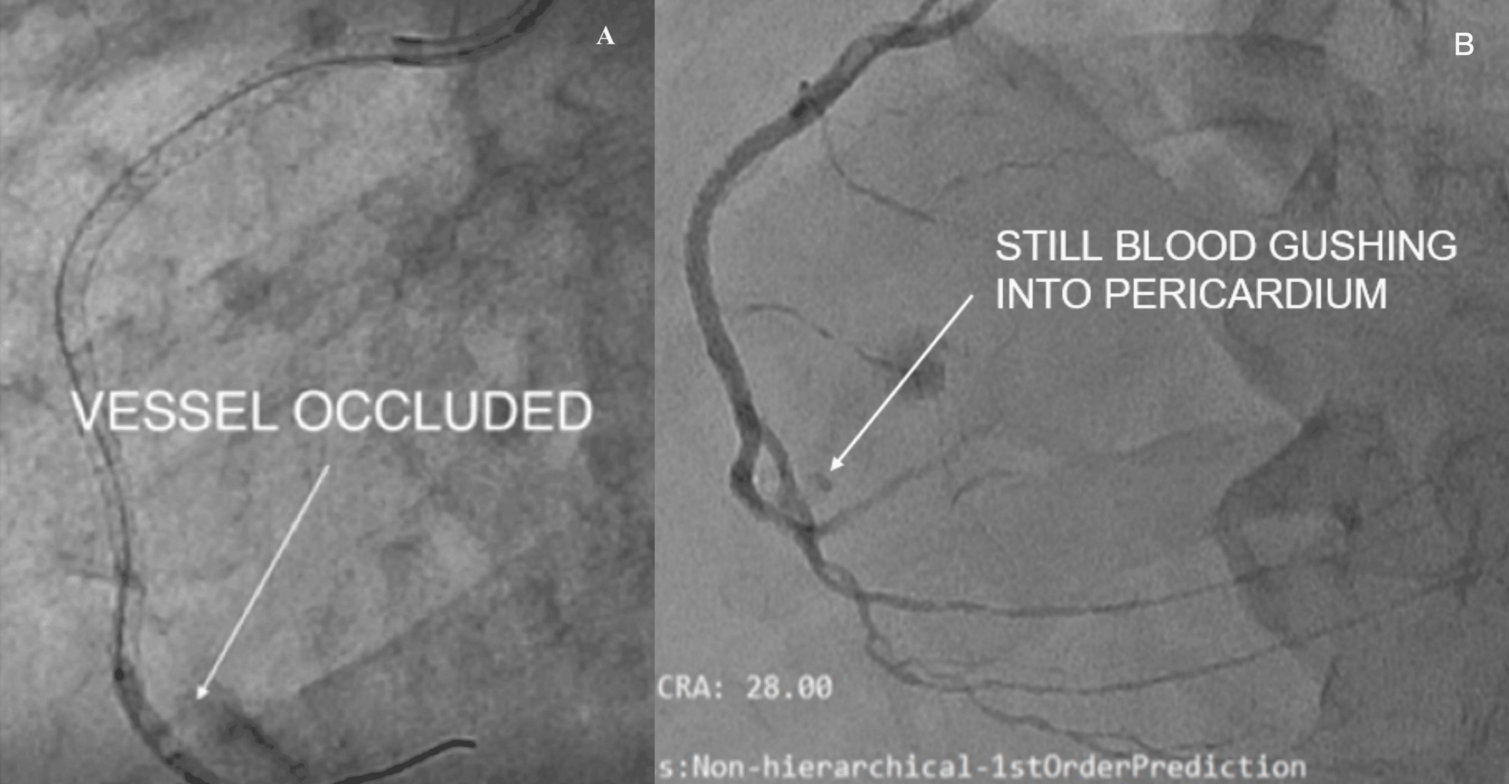
Post-balloon occlusion, there was a persistent contrast blush, suggesting a failed maneuver A: immediate balloon tamponade was done to prevent the hemodynamic compromise (white arrow); B: despite the initial conservative procedure, there was still a persistent contrast blush into the pericardium (white arrow).

Prolonged balloon tamponade was performed, along with reversal of anticoagulation [7]; however, these measures failed to seal the perforation and arrest the contrast leak (Figure 7).

Definitive management strategies

An attempt was made to deliver a Graftmaster-covered stent (Abbott Vascular, IL, USA) [8,9] to the site of perforation, but advancement was unsuccessful due to vessel tortuosity and complex anatomy.

Novel intervention

**Figure 8:**
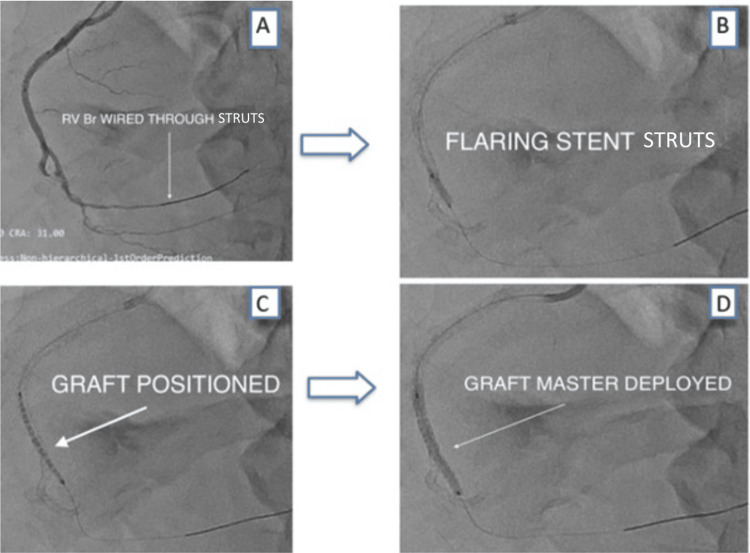
Targeted vessel annulment was done by redirecting the flow to the marginal branch A, B: guidewire was manipulated through the struts of the deployed drug-eluting stent (DES) (white arrow); C: the cover Graftmaster stent was positioned to annul the bleeding territory (white arrow); D: Graftmaster stent was deployed and fixed into the acute marginal branch (white arrow).

A second guidewire was advanced into the marginal branch through the struts of the previously deployed stent. The stent struts were then dilated using a 2.0 × 12 mm balloon, which facilitated the successful delivery of a 2.8 × 26 mm Graftmaster stent at 18 atm. This innovative maneuver redirected blood flow from the RCA ostium into the marginal branch, functionally excluding the distal perforated RCA segment. The perforation was thereby sealed, effectively controlling bleeding and stabilizing the patient (Figure 8).

Post-procedural course and outcome

**Figure 9:**
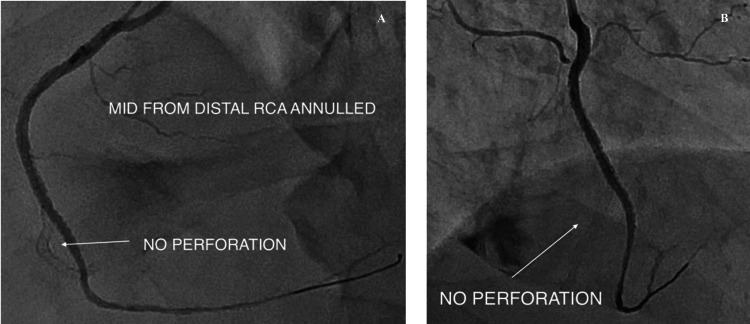
Final angiogram From the mid to distal right coronary artery is annulled, and there is active contrast blush in the pericardium. A, B: final check angiogram revealed no contrast blush, and, white arrow shows the annulled segment of the vessel that was perforated.

Angiography following stent deployment showed complete stoppage of contrast extravasation, with restoration of stable flow into the marginal branch and no further leakage from the distal RCA (Figure 9). The patient's hemodynamic status improved immediately, with no pericardial effusion seen on subsequent echocardiography.

He was admitted to the coronary care unit, where serial echocardiograms demonstrated no pericardial tamponade and normal ventricular function. Cardiac enzymes remained within appropriate limits, and ECG monitoring showed no new ischemic changes.

The patient was discharged in a stable condition on dual antiplatelet therapy and appropriate secondary prevention medications. At one month of follow-up, he remained asymptomatic with no evidence of recurrent ischemia or pericardial effusion on clinical and echocardiographic evaluation.

## Discussion

CTO-PCI demands advanced operator skill due to the complexity of lesion morphology and limited distal flow. Wire escalation techniques and the use of microcatheters are standard for navigating complex occlusions. In this case, overlapping DES allowed full lesion coverage, but a rare complication, distal vessel perforation, occurred, underscoring the procedural risk.

Perforations are uncommon (0.1-0.5% in general PCI; up to 4% in CTO) [12], but when they occur, they necessitate immediate action. Usually managed by prolonged proximal balloon inflation for around 30-60 minutes, usage of anticoagulant correction therapy like protamine can be tried, and a cover stent can be placed generally in a proximal vessel perforation of >2.75 mm, or many other techniques like the use of coiling, microspheres (non-absorbable, hydrophilic particles), autologous blood clots, and fat embolization are tried with less success [12-15]. Covered stents like Graftmaster are the treatment of choice for large perforations, but their use is limited in tortuous vessels due to size and rigidity. Table 1 summarizes the management modalities and outcomes in large CAP studies.

**Table 1:** Management modalities and outcomes in large CAP studies CAP: coronary artery perforation

Management Strategy	Usage Rate (%)	Success Rate (%)	Key Outcomes/Notes	Citations
Balloon Tamponade	29–59	54-89	First-line for many cases	Mikhail et al., 2022; Al-Lamee et al., 2011; Krishnegowda et al., 2020 [2,6,7]
Covered Stent	24-67	84-96	Mainstay for large vessel CAP	Mikhail et al., 2022; Voll et al., 2025; Al-Lamee et al., 2011; Krishnegowda et al., 2020 [2,6-8]
Coil/Fat Embolization	<10	No significant data available	For distal/collateral perforations	Abdalwahab et al., 2020; Al-Lamee et al., 2011; Krishnegowda et al., 2020 [3,6,7]
Surgical Intervention	12-21	44–100	For refractory/unstable cases	Mikhail et al., 2022; Lemmert et al., 2017; Al-Lamee et al., 2011; Krishnegowda et al., 2020 [2,5-7]
Conservative Management	10-27	High (minor CAP)	For minor, hemodynamically stable cases	Lemmert et al., 2017; Abdalwahab et al., 2020; Krishnegowda et al., 2020 [5,3,7]

Vessel annulment was done in this case, which also had an adequate collateral blood supply for the segment. Studies have shown that collateral connection grade 2 (CC2), which are already well-perfusing collaterals, will protect the myocardium in the case of an acute re-occlusion [9].

**Figure 10:**
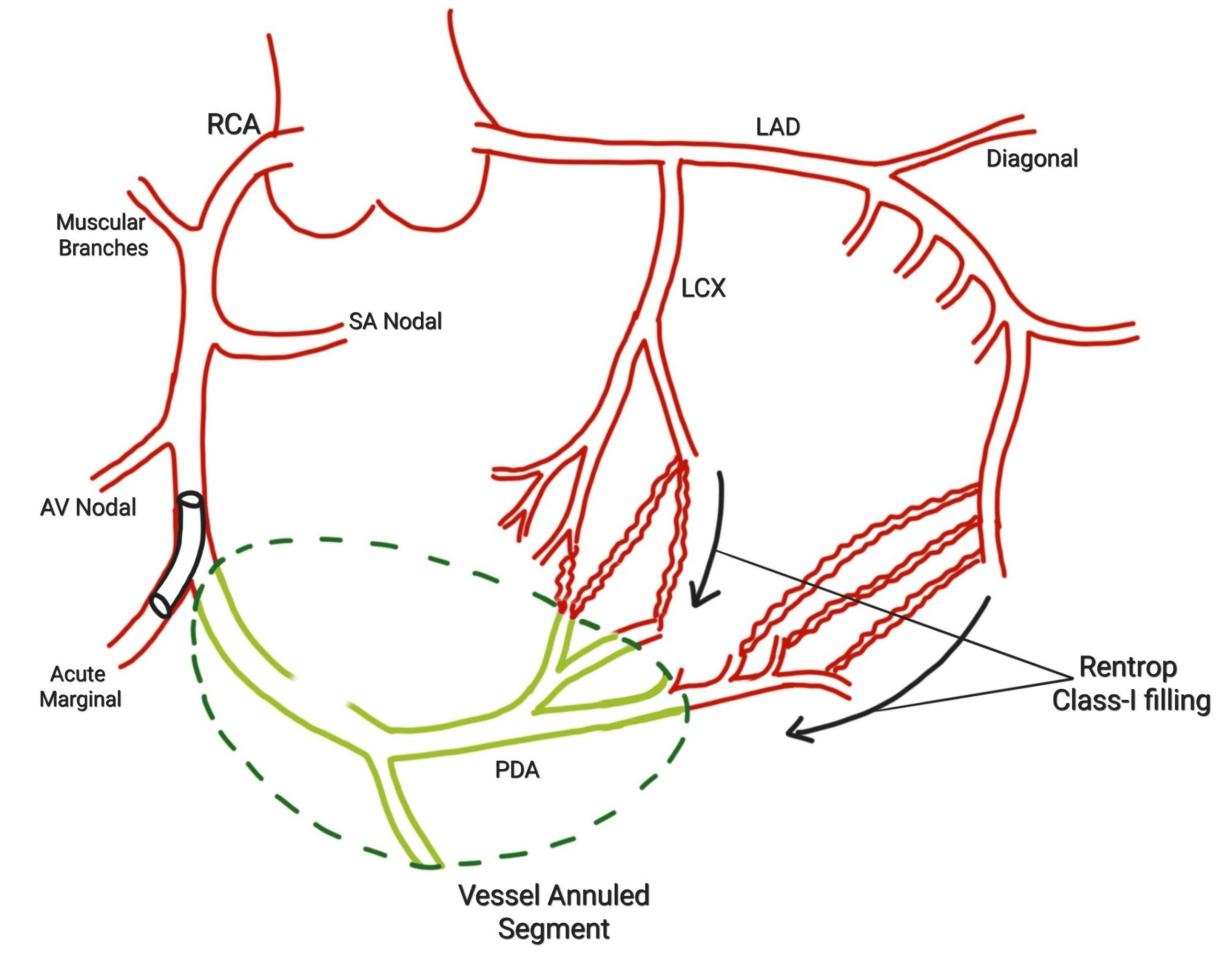
This schematic diagram shows where the vessel's annulated segment (green) is perfused with CC2 collaterals RCA: right coronary artery; LAD: left anterior descending; LCX: left circumflex; PDA: posterior descending artery; CC2: collateral connection grade 2; AV: atrio-ventricular; SA: sino-atrial Figure created by the author, Ojas Gowda S G

In our case, conventional access failed. Through creative intervention, stent-strut dilatation, and alternate vessel access, we successfully delivered a covered stent via the marginal branch to seal the perforation and exclude the damaged RCA segment (Figure 10). This represents a novel, life-saving bailout technique not previously documented in literature. It reinforces the importance of flexibility and innovation during high-risk procedures.

Limitations

The limitations of this case are mentioned in Table 2.

**Table 2:** Limitations of the case report RCA: right coronary artery

No.	Limitation	Explanation/Implication
1	Loss of distal RCA perfusion	Functional exclusion of the distal RCA may compromise myocardial perfusion in the affected territory, potentially leading to ischemia if collateral supply is inadequate.
2	Uncertain long-term outcomes	The long-term patency, structural stability, and endothelialization of a covered stent deployed through side-branch struts remain unknown and may predispose to restenosis or stent thrombosis.
3	Technical complexity/reproducibility	The procedure requires advanced operator skill, precise wire manipulation, and imaging guidance, which may limit its feasibility in centers with less interventional experience.
4	Off-label technique	Deployment of a covered stent through stent struts is an unconventional, off-label approach with limited literature support and an undefined safety profile.
5	Reserved for bailout scenarios	This method should be considered only when conventional strategies are unsuccessful or technically unfeasible.

## Conclusions

This case demonstrates a unique and effective bailout strategy in the management of life-threatening distal RCA perforation during complex CTO-PCI. After the failure of conventional measures, a well-timed and innovative approach, deploying a covered stent through the marginal branch to functionally exclude the perforated segment, successfully sealed the leak and stabilized the patient. Rentrop grade 1 collateral flow from the LAD minimized ischemic consequences following distal vessel occlusion. Several important clinical lessons underpin this experience: early recognition and swift action are crucial for coronary perforations; when standard techniques fail, flexibility is required within procedures; and individual decision-making must be guided by coronary anatomy and collateral circulation. He was symptom-free at the 12-month follow-up. Ultimately, this case reaffirms that timely judgment and adaptability in the catheterization laboratory are critical to achieving favorable outcomes in catastrophic PCI complications.
